# Cancer mortality in 2020 and its trend analysis in Inner Mongolia during four time periods from 1973 to 2020

**DOI:** 10.3389/fonc.2023.1096968

**Published:** 2023-01-31

**Authors:** Ruili Hou, Zhiqi Mu, Weiwei Kang, Zhengran Liu, Buqi Na, Wenliang Niu

**Affiliations:** ^1^ School of Public Health, Baotou Medical College, Baotou, China; ^2^ Institute of Nutrition and Food and Health, Baotou Medical College, Baotou, China; ^3^ Department of Psychiatric Prevention and Control, Heping District Center for Disease Control and Prevention, Shenyang, China; ^4^ Department of Chronic Noncommunicable Diseases Prevention and Control, The Inner Mongolia Autonomous Region Comprehensive Center for Disease Control and Prevention, Hohhot, China; ^5^ Neurology Department, The First Affiliated Hospital of Baotou Medical College, Baotou, China

**Keywords:** cancer, mortality rate, APC, trend, Inner Mongolia

## Abstract

Cancer is one of the leading causes of mortality in China and is responsible for placing a major burden on its economic system. Inner Mongolia is located close to the northern border of China and spans more than 2,400 km from east to west. It has a total area of 1,183,000 km^2^, which accounts for about one-third of the total area of the country. Its ethnic demographics are complex and unique. We were interested in understanding whether these situations lead to a higher mortality rate for certain types of cancer, which was the motivation behind our research. This study aims to estimate cancer mortality in Inner Mongolia, its burden, and its trend over a 60-year time span. We compiled data pertaining to cancer in Inner Mongolia, obtained from the three national causes of death sample surveys. In addition, we obtained data pertaining to cancer mortality rates from the cause of death surveillance system in Inner Mongolia in the year 2020. The proportion of deaths from various cancers, crude mortality rates, and standardized mortality rates were calculated. The Joinpoint Regression Program was used to calculate mortality trends and the periodic percentage change (PPC) in these rates. We found that the mortality rate of cancer was 142.15/10^5^; the age-standardized mortality rate using the Chinese standard population in 2000 (ASMRC) and Segi’s world population (ASMRW) were 86.49/10^5^ and 85.73/10^5^ in 2020, respectively. From 1973 to 2020, during the four time periods, the leading five cancer types contributing to the mortality rate among Inner Mongolia residents in 2020 were stomach cancer, esophageal cancer, liver cancer, cervix/uterine cancer, and lung cancer. The four periods of time PYLL ‰ were 9.05‰, 10.79‰, 12.1‰, and 10.38‰ from 1973 to 2020. The overall trend of the crude mortality rate of cancer in Inner Mongolia from 1973 to 2020 increased (PPC =1.77%, P<0.05). Also, the crude mortality rate and ASMRC were higher in men than in women (P<0.05). The mortality rates of cancer in Inner Mongolia increased with age ie first time period and in 2020 for ages 10 to 84. The same trend was observed for ages 0 and 74 in the second and the third time periods. Overall, in 2020, the CMR and the ASMRW in Inner Mongolia were lower than the national level and significantly lower than those in other domestic Chinese provinces. Lung cancer was the most reported cancer. Cancers from the first to seventh place ranking were consistent with the national ranking in 2020. The overall crude mortality rate of cancer in Inner Mongolia during the four periods revealed an increasing trend, and liver cancer-related mortality also showed an upward trend during the four periods. The findings may provide baseline data for cancer research.

## Introduction

Cancer is the second-leading cause of death worldwide and imposes a significant burden on the public health system and the economic system (1–4). The rapid increase in cancer incidence and mortality worldwide is not only related to an aging population and the increase in population size but also reflects changes in the prevalence of the main risk factors for cancer and their distribution (of the main risk factors). This study focused on two factors: age and sex. China has a large population, and cancer mortality rates are relatively high in China (5, 6). Cancer-related deaths could increase by 207,101 from 2020 to 2022 (6, 7). Inner Mongolia has a large east-west span (183,000 km^2^), accounting for about one-third of the total area of the country. Inner Mongolia is adjacent to Mongolia and Russia and consists of plateaus, mountains, hills, plains, deserts, rivers, lakes, and other landscapes. It has a temperate continental climate. Evidently, the composition of the nation is complex. However, we are uncertain if these complications affect the rate of cancer mortality in Inner Mongolia or whether its disease burden is severe. We also cannot be certain how the cancer mortality rate has changed in the last 60 years. Several studies were conducted over a large time span, some more than 60 years ago; however, they were conducted at a national level (3, 8). Furthermore, some published research estimates of mortality rates only focus on specific types of cancer in Inner Mongolia; moreover, other reported estimates of mortality only use data from a single year or even a shorter time (9–11).

The current study aimed to estimate cancer mortality, its burden, and its trend over a 60-year time span in Inner Mongolia. Some research suggests that cancer control efforts must contend with the issue of geographical disparity. This may offer cancer researchers some basic data and provide a reference for Mongolia and Russia.

## Data and methods

### Data sources

We retrospectively analyzed the data gathered from Inner Mongolia, which were extracted from the three national reviews of the cause of death for the periods between 1973–1975 (the first time period), 1990–1992 (the second time period), and 2004–2005 (the third time period). The three national reviews of the cause of death survey were organized by the National Cancer Control Office. This is a nationwide retrospective survey of cancer mortality that involves the active participation of the public and of local health departments at all levels. Retrospective multistage stratified random whole-group samplings related to causes of death covering 1/10 of the population nationwide were conducted. The surveys collected data pertaining to causes of death and relevant demographic, socioeconomic, cultural, and health service-related data during these periods (12, 13). The quality of this survey was considered good.

The recently registered data were extracted from the Cause of Death Registration Information System in China in 2020 and population data from the Integrated Management System of the Chinese Center for Disease Control and Prevention (CDC). The cause of death registration in Inner Mongolia covers 103 regions and counties, with a total of 105 monitoring sites. Data from 72 monitoring points with a crude death rate greater than 5‰ in the region were used for analysis, including 48 urban and 24 rural monitoring points. The coverage of the population accounted for 67.78% of the region, including 33.69% of the urban population and 66.31% of the rural population, with a male-to-female ratio of 105:100. The mortality of cancer data in China, were released by the International Agency for Research on Cancer (gco.iarc.fr).

### Statistical analysis

Statistical analysis was performed to calculate the CMR (crude mortality rate), standardized mortality, in which standardized mortality included ASMRC (age-standardized mortality rate, based on the Chinese standard population in 2000) (14) as well as ASMRW (age-standardized mortality rate, based on Segi’s world population), potential years of life lost (PYLL), and PYLL‰, where PYLL represents the cumulative number of years of life lost in a population due to “premature death” (15).

R 4.2.1 was used for statistical analysis, and differences in CMR, ASMRC, and PYLL‰ between sexes were assessed with the analysis of the Chi-square test. The trend Chi-square test was applied to age trend analysis. Periodical percentage change (PPC) of mortality and PYLL‰ was calculated using the Joinpoint Regression Program [version 4.9.1.0; Joinpoint Regression Program (www.cancer.gov)]. Joinpoint regression analysis uses a piecewise linear regression method to determine the trend displayed by one or more line segments (16). Joinpoint regression models include linear models (y = xb) and log-linear models (lny = xb). Linear models were chosen if the dependent variable (y) follows a normal (or approximately normal) distribution and the data sample size is large (usually greater than 100), while log-linear models were preferred if the dependent variable follows an exponential or Poisson distribution. Log-linear models are generally chosen when analyzing population-based trends in tumor mortality models; a log-linear model was chosen for this study (17). PPC was the average period percentage change in the dependent variable. Significant changes include changes in the direction or in the rate of increase or decrease. A P-value of less than 0.05 was considered statistically significant (18).

PYLL is calculated by the formula $PYLL=\sum_{i\;=\;1}^{n}\left[\left(L-X_{i}\right)\times d_{i}\right]$ where *L* represents life expectancy (70 years in this study) (19), *X*_*i*_ is the median of an age group, and *d*_*i*_ is the number of deaths in an age group.

PYLL‰ is calculated by the formula *PYLL*‰=*PYLL*/*N*×1,000‰ where *N* represents the population size.

Regression model: ln (y)=α + *β*X + *ϵ*

Where y represents the mortality rate or PYLL‰, α represents the constant term, *β* represents the regression coefficient, and *ϵ* represents the random error.

PPC was estimated using the regression coefficient *β*.


$$\text{PPC}=(e^{\beta }-1)\;\times \;100\%$$

Where e represents the base of the Natural Logarithms, which is a mathematical constant that is approximately equal to 2.71828.

## Results

### Cancer mortality in 2020

In 2020, 17,211,970 residents were recorded in the registration areas of Inner Mongolia. The region’s mortality rate contributed by malignant tumors was 142.15/10^5^ (179.17/10^5^ for males and 103.20/100,000/10^5^ for females). ASMRC was 86.49/10^5^ (113.65/10^5^ for males and 60.69/10^5^ for females), and ASMRW was 85.73/10^5^ (113.56/10^5^ for males and 59.33/10^5^ for females). The top 10 origins of cancer that resulted in death were the lungs, liver, stomach, esophagus, colorectum, pancreas, breast, leukemia, lymphoma and multiple myeloma, lips, the oral cavity, and the pharynx.

### Profiles of cancer deaths in the four periods

**Figure 1:**
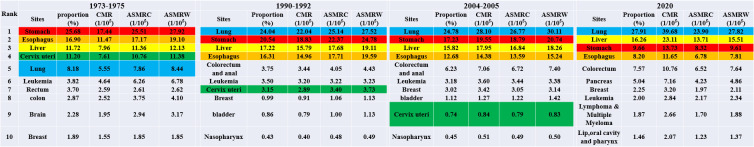
The top 10 cancer rank during the four periods in Inner Mongolia.

The leading 10 types of cancer that resulted in deaths among Inner Mongolian residents during the four periods are shown in **Figure 1**. It was estimated that during the first time period, the top five cancers were stomach, esophageal, liver, cervix/uterus, and lung cancer; during the second time period, the top five cancers were lung, stomach, liver, esophageal, colorectal, and anal cancer; and in the third time period, the top five cancers were lung, stomach, liver, esophageal, colorectal, and anal cancer; lung, liver, stomach, esophageal, and colorectum cancer were the top cancers in 2020. Among them, lung cancer rose from the fifth most common cause of cancer-related mortality in the first time period to the most common cause in the second time period; moreover, it remained the most common cause in the third time period and in 2020. Its proportion increased during the four periods, but the mortality rate increased except in 2020 (PPC = 4.29, *P* = 0.06). From the first time period to the second time period, lung cancer mortality varied the most, with an ASMRW increase of 19.38%; cervix/uterus cancer fell from fourth place in the first time period to seventh place in the second time period and further dropped to ninth place in the third time period and was ultimately removed from the list of the top ten causes in 2020; furthermore, mortality decreased. The proportion of deaths related to stomach cancer decreased and fell from the topmost common cause in the first time period to second place in the second time period, remained in second place in the third time period, but fell to third place in 2020 (PPC = −0.43, *P* = 0.48). Liver cancer-related mortality remained in third place during the three reviews but rose to second place in 2020 (PPC = 2.29, *P* = 0.04). The proportion of mortality fluctuated but remained relatively large. The death rate of esophageal cancer did not significantly change between the first time period and the second time period, coming in at 19.10/10^5^ and 19.59/10^5^, respectively, but started to decline in the third time period, dropping to 7.81/10^5^ by 2020 (PPC = 0.04, *P* = 0.95).

### Cancer mortality by sex

**Figure 2:**
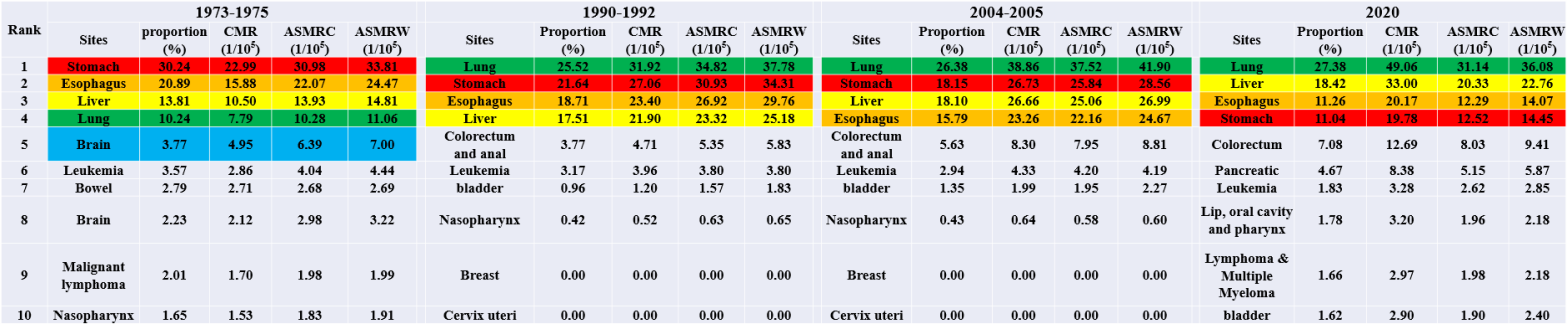
The top 10 cancer rank in male during the four periods in Inner Mongolia.

The top ten cancers with a high mortality rate in males in Inner Mongolia during the four periods are shown in **Figure 2**. Stomach, esophageal, liver, and lung cancer were always in the top five during the four periods. Like the mortality trends observed in both males and females, lung cancer rose from fourth place in the first time period to first place in the second time period. It maintained first place in the third time period and in 2020, and the proportion increased during the four periods. Notably, however, the mortality rate increased, except in 2020, where it dropped. The proportion of deaths from gastric cancer decreased from the most common cause in the first time period to the second most common cause in the second time period, remained the same in the third time period, but subsequently dropped to the fourth most common cause in 2020. Liver cancer-related mortality in males showed an upward trend during the four periods (PPC = 2.51, P = 0.04).

**Figure 3:**
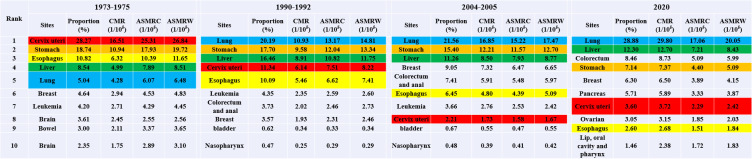
The top 10 cancer rank in female during the four periods in Inner Mongolia.

The top ten cancers with high mortality in females in Inner Mongolia during the four periods are shown in **Figure 3**. Cervical/uterine cancer ranked first in deaths in the first time period. The mortality of cervical cancer and associated mortality rates declined during the three national reviews on cause of death but slightly rebounded in 2020. Lung cancer mortality continued to increase, moving from the fourth position in the first time period to the first position in the second time period and maintaining this position in the third time period and 2020 (PPC = 4.31, P = 0.01). The mortality rate of breast cancer showed fractal fluctuations in these four time periods, with an ASMRW of 4.831/10^5^, 2.461/10^5^, 6.651/10^5^, and 4.151/10^5^, respectively. The ASMRC and ASMRW of esophageal cancer showed a decreasing trend (PPC = −4.03, P = 0.04; PPC = −3.85, P = 0.04).

### Trends of mortality and PYLL‰ during the four periods

**Figure 4:**
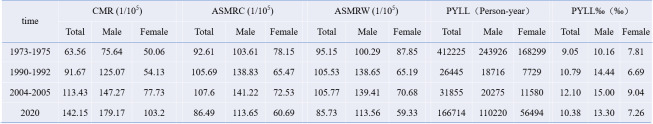
Mortality of cancers in Inner Mongolia, four times in the period of 1973–2020, Chi-square values of crude mortality between different sexes in the four period were 279.201, 346.055, 292.948, and 1,748.216, respectively; Chi-square values of ASMRC between different sexes were 1,393,0071.440, 1,779,283.759, 1,846,753.674, and 11,412,456.166, respectively; Chi-square values of the PYLL‰ between different sexes were 826.471, 3,430.695, 1,952.916, and 14,262.834, respectively. All P-values were less than 0.05.

The four time periods of PYLL‰ were 9.05‰, 10.79‰, 12.1‰, and 10.38‰ from 1973 to 2020. The overall crude mortality rate of cancer in Inner Mongolia during the four periods revealed an increasing trend (PPC = 1.77%, *P<*0.05), including an increasing trend in the crude mortality rate in males (PPC = 1.86%, *P<*0.05) and an increasing trend in the crude mortality rate in females (PPC = 1.68%, *P<*0.05). The overall standardized mortality rate of cancer showed a decreasing trend (PPC = −0.10%, *P* = 0.81) with an increasing trend for men (PPC = 0.23%, *P* = 0.71) and a decreasing trend for women (PPC = −0.45%, *P* = 0.23), but there were no trend changes that were statistically significant. The overall trend of PYLL% in cancer was increasing (PPC = 0.37%, *P* = 0.40), with an increasing trend in both males (PPC = 0.60%, *P* = 0.34) and females (PPC = 0.46%, *P* = 0.93), but none of the trend changes were statistically significant. Crude mortality, standardized mortality, and PYLL% were higher in males than in females (**Figure 4**).

### Age-specific mortality rates and trends of cancer

**Figure 5:**
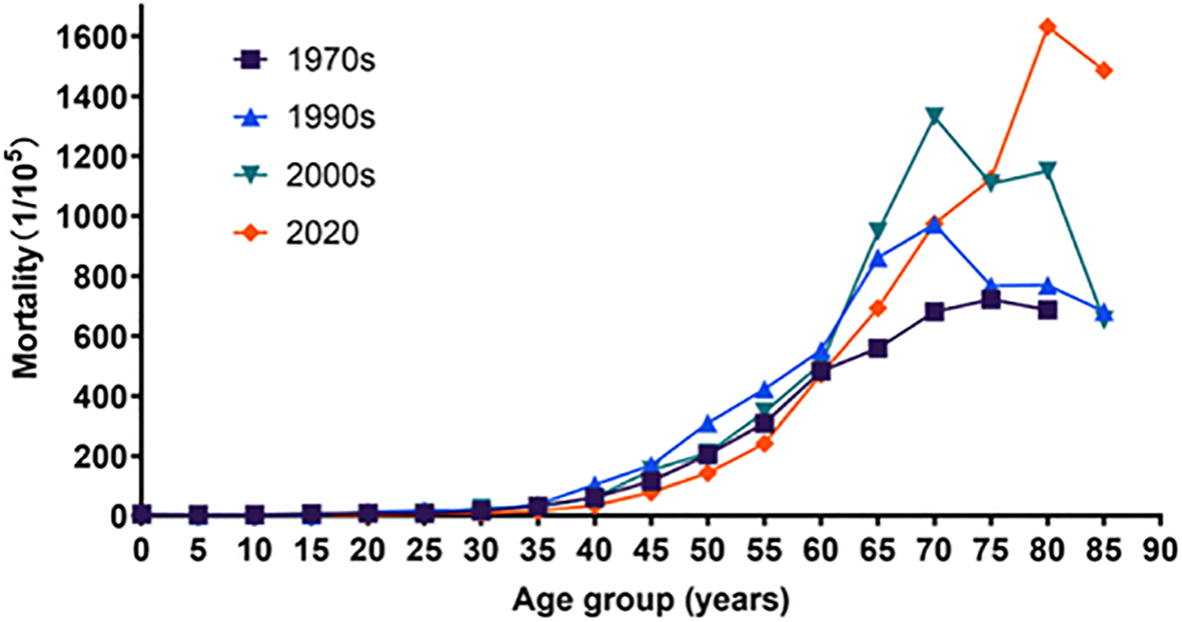
Age-specific mortality rates and trends of cancer, trends of Chi-square of the first time, the second time, the third time, and 2020 were 47,628.486, 5,696.633, 6,482.973, and 0957.890, respectively. All P-values were less than 0.05.

The mortality rates of cancer in Inner Mongolia increased with age in the first time period and in 2020 for ages 10 to 84. The same trend was found for ages 0 to 74 in the second time period and the third time period. After peaking at age 75, it began to decline. The mortality rates for ages 0–64 in 2020 were lower than the previous three reported values (**Table 1**; **Figure 5**).

**Table 1:** Age-specific mortality rates of cancer during four periods (1/10^5^).

Age group (years)	1973–1975	1990–1992	2004–2005	2020
0∼	5.75	4.49	3.37	3.20
5∼	4.84	1.65	1.93	2.68
10∼	4.17	3.26	2.30	2.69
15∼	7.18	3.87	6.72	3.70
20∼	8.11	9.55	8.89	2.86
25∼	7.84	11.56	7.96	6.72
30∼	17.09	15.68	22.98	8.87
35∼	35.07	32.78	32.87	15.75
40∼	64.87	80.56	54.75	30.15
45∼	123.47	126.17	108.84	63.08
50∼	209.90	222.34	157.53	106.32
55∼	291.54	322.92	263.62	173.19
60∼	437.40	452.38	378.52	346.14
65∼	515.36	619.54	724.12	506.30
70∼	617.48	742.59	934.20	711.70
75∼	636.92	612.07	894.33	869.64
80∼	682.84	575.45	857.26	1,387.63
85+	–	524.68	529.70	1,103.32

The last age group was set to 80+ for the period 1973–1975 and was not further subdivided. + means age > 85 years, -means the data set is missing, ∼ means from the lower limit of this group segment to the lower limit of the next group segment, but does not include the lower limit of the next group segment, e.g., 65~ means from age 65 to 69.

### Age-specific mortality rates and trends by sex

**Figure 6:**
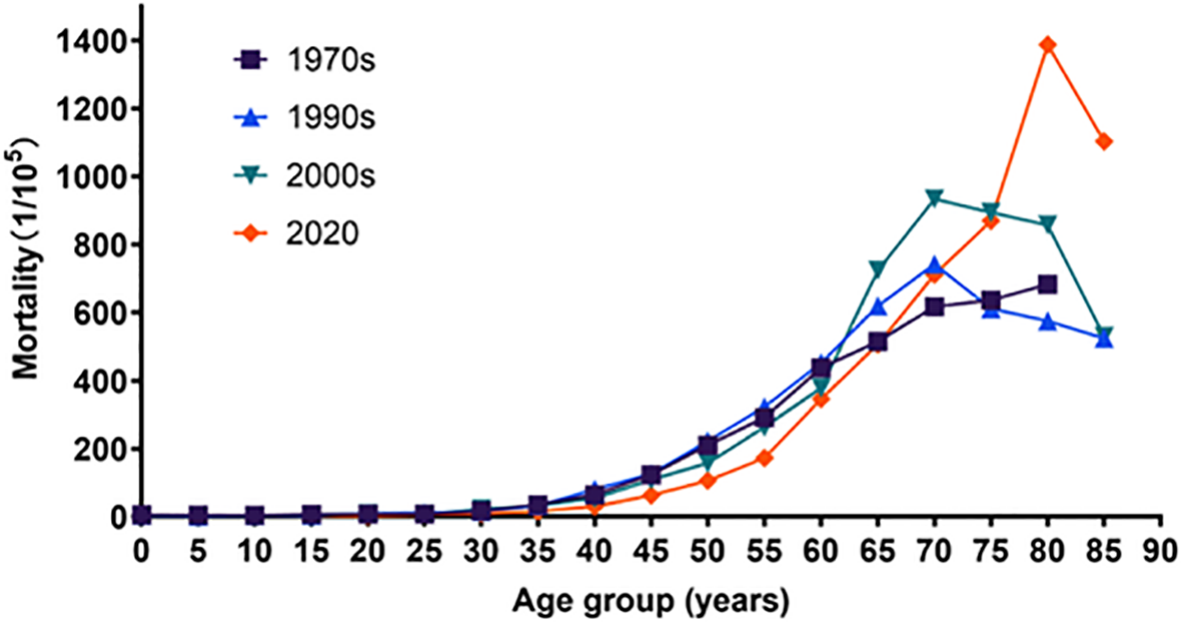
Age-specific mortality rates and trends in male, trends of Chi-square of the 1970s, the 1990s, the 2000s, and 2020 were 28,189.370, 3,971.809, 4,486.643, and 27,102.434, respectively. All P-values were less than 0.05.

**Figure 7:**
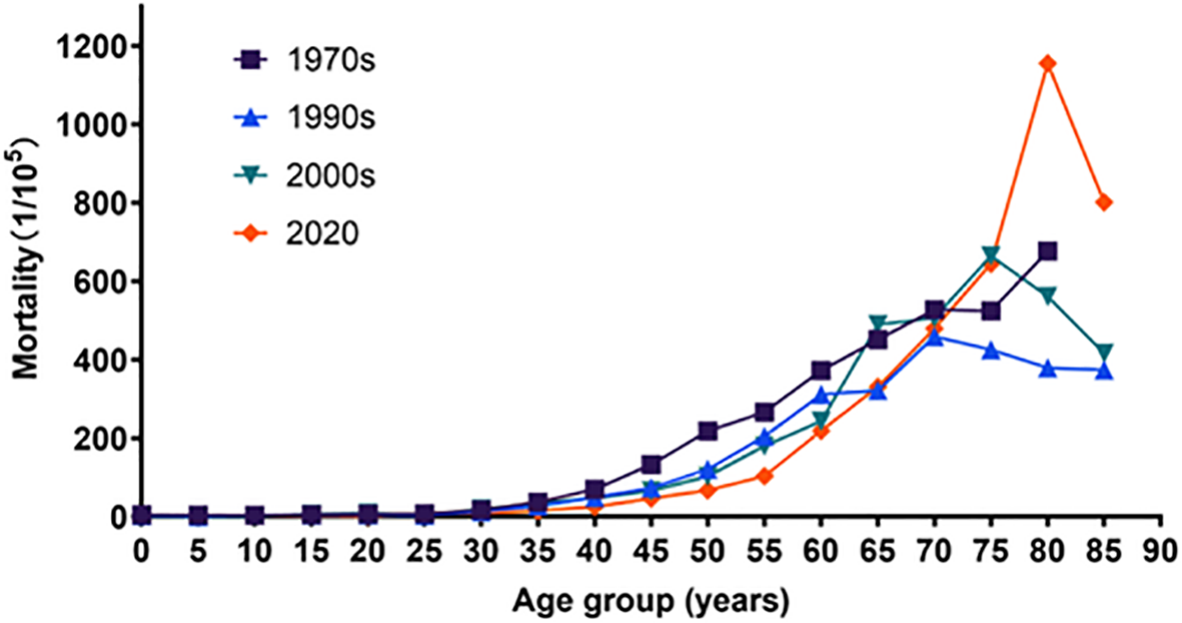
Age-specific mortality rates of cancer during four periods in female, trends of Chi-square of the 1970s, the 1990s, the 2000s, and 2002 were 19,043.474, 1,633.955, 2,013.222, and 14,508.884, respectively. All P-values were less than 0.05.

The mortality rates of cancer in Inner Mongolia increased before age 75 during the four periods in both males and females. After peaking at age 75, it began to decline in the second and the third time periods in males; however, differences in mortality appeared in 2020, and the mortality rate decreased over age 85. In females, the trend of mortality rates was similar to that in the first time period and in the second time period. While this trend peaked at the age of 70–74, the peak in mortality was delayed by 5 years in the third time period. In 2020, the mortality rate did not begin to decline until age 85. The mortality rates for cancer for ages 0–64 in 2020 are smaller than the three previous data groups. In contrast, mortality rates after age 65 gradually exceeded the other three data groups and fully exceeded the other three data groups after age 80 (**Tables 2**, **3** **;** **Figures 6**, **7**).

**Table 2:** Age-specific mortality rates of cancer during four periods in male (1/10^5^).

Age group (years)	1973–1975	1990–1992	2004–2005	2020
0∼	6.27	5.65	4.55	3.67
5∼	5.21	2.35	3.57	3.07
10∼	4.39	3.16	3.61	3.40
15∼	8.34	3.44	6.68	4.92
20∼	9.09	13.72	8.92	4.32
25∼	8.02	18.45	9.97	6.67
30∼	16.30	18.23	25.89	8.57
35∼	32.95	38.15	30.65	16.32
40∼	60.77	104.96	61.34	34.60
45∼	116.09	168.79	152.31	78.54
50∼	203.76	309.66	210.28	143.78
55∼	309.13	422.98	347.05	242.07
60∼	483.07	552.97	507.56	472.38
65∼	559.34	861.76	948.07	693.45
70∼	681.38	972.93	1,331.24	974.97
75∼	722.11	768.20	1,108.19	1,124.12
80∼	687.61	769.38	1,150.24	1,631.76
85+	–	681.60	653.00	1,485.98

+ means age > 85 years, -means the data set is missing, ∼ means from the lower limit of this group segment to the lower limit of the next group segment, but does not include the lower limit of the next group segment, e.g., 65~ means from age 65 to 69.

**Table 3:** Age-specific mortality rates of cancer during four periods in female (1/10^5^).

Age group (years)	1973–1975	1990–1992	2004–2005	2020
0∼	5.19	3.18	1.89	2.72
5**∼**	4.43	0.87	0.00	2.25
10**∼**	3.92	3.37	0.94	1.89
15**∼**	5.84	4.31	6.77	2.34
20**∼**	7.03	5.00	8.87	1.21
25**∼**	7.64	3.90	5.91	6.77
30**∼**	17.95	12.91	19.93	9.20
35**∼**	37.48	27.01	35.37	15.12
40**∼**	69.96	49.57	47.55	25.34
45**∼**	133.31	73.29	65.88	46.35
50**∼**	218.15	120.75	103.20	67.08
55**∼**	266.90	205.09	179.86	103.54
60**∼**	372.90	311.34	243.78	219.23
65**∼**	451.27	321.63	489.03	331.11
70**∼**	527.37	459.31	507.05	479.03
75**∼**	524.55	425.61	664.56	643.99
80**∼**	676.71	379.24	561.05	1,154.52
85+	–	374.01	416.46	801.23

+ means age > 85 years, -means the data set is missing, ∼ means from the lower limit of this group segment to the lower limit of the next group segment, but does not include the lower limit of the next group segment, e.g., 65~ means from age 65 to 69.

## Discussion

In 2020, the CMR of cancer was 142.15/10^5^ in Inner Mongolia, and the ASMRW was 85.73/10^5^, which was lower than the national level (the CMR of cancer was 207.5/10^5^, and the ASMRW was 129.4/10^5^). The ASMRW was lower than that of the United States (86.30/10^5^) and the United Kingdom (100.50/10^5^), and significantly lower than other domestic Chinese provinces, such as Qinghai Province, the Ningxia Autonomous Region, and Shanxi Province (20–22). The top 10 cancer-related deaths in Inner Mongolia were related to the lungs, liver, stomach, esophagus, colorectal region, pancreas, breast, bone marrow (leukemia), lip, oral cavity, pharynx, lymphoma, and multiple myeloma. This accounts for 82.22% of all causes of death. Regardless of sex, lung cancer is the most reported cancer. Cancers from the first to seventh place ranking were consistent with the national ranking in 2020. The least three most common cancers reported differed from what was reported in China, namely brain cancer, central nervous system cancer, and leukemia and cervix/uterus in China (23). In Inner Mongolia, the ASMRW of the top 10 cancers was all lower than that in China. It might be associated with increased public awareness of cancer prevention following governmental efforts to educate the public about cancer education (24).

In 2020, the cancer rates among male residents (CMR and ASMRW were 179.17/10^5^ and 113.56/10^5^, respectively) were lower than the national level (CMR and ASMRW were 245.3/10^5^ and 163.9/10^5^, respectively). In 2020, the cancer rates among female residents (CMR and ASMRW were 103.20/10^5^ and 59.33/10^5^, respectively) were still lower than the national level (CMR and ASMRW were 167.7/10^5^ and 98.1/10^5^). The CMR, ASMRC, ASMRW, PYLL, and PYLL‰ rates of cancer in Inner Mongolia residents were higher in men than in women during the four reported periods, which is consistent with national findings (25). Global Cancer Statistics 2020 showed that the male cancer death rate is 43% higher than that of women (5). The ASMRW in our study was approximately twice as high in men as in women. Other published research is also consistent with our findings (26). The sex differences in cancer mortality may be caused by different working environments exposed to different carcinogenic factors, and of course, some scholars believe that different hormone levels affect the metabolism of carcinogens and thus lead to the occurrence of cancer (27).

This study found that the top five cancers causing deaths in Inner Mongolia in the first time period affected the stomach, esophagus, liver, cervix/uterus, and lung; in the second time period, lung, stomach, liver, esophagus, and the colon/anus; and in the third time period, lung, stomach, liver, esophagus, and colon/anus cancer. The top five cancer-related causes of death account for approximately 70% of all cancer deaths. From the second time period up to 2020, the top three cancers reported were lung cancer, liver cancer, and stomach cancer. We speculate that these three types of cancer may remain the main forms of cancer that contribute to death among Inner Mongolian residents in the future, which is consistent with the national data in Zongchao’s interpretation of the report (6). Lung cancer rose from fifth place in the first time period to first place in the second time period. It subsequently maintained first place in the third time period and until 2020. Moreover, the proportion and CMR increased during the four periods, but the ASMRW still increased except in 2020. From the first to the second time period, lung cancer mortality varied the most, with an ASMRW increase of 19.38%. Some studies suggested that this was due to the rapid growth of the economy, leading to industrial development and rapid urbanization, resulting in environmental degradation. Other major contributors include a high rate of smoking among residents, second-hand smoking, air pollution, and inhalation of grease smoke (from long-term cooking) (28). The fact that the ASMRW for lung cancer in 2020 is a little lower than it was in the third time period could be attributed to Inner Mongolia’s ageing population. The figures show that overall, there were 860,000 elderly individuals in the fourth census, 1.29 million in the fifth, and 1.88 million in the sixth. The average growth rate in Inner Mongolia is 47.5%, and the elderly population of Inner Mongolia is growing quickly (29). Additionally, this explains why standard cancer mortality is lower than crude mortality. Therefore, elderly people should be the target group for cancer prevention and treatment (30). Stomach, esophageal, and liver cancers were also commonly diagnosed and identified as leading causes of cancer death (3). Although stomach cancer was still one of the cancer types with a high death rate (31), its ASMRW did slowly decrease during the four periods in our study, which is consistent with the study by Cao (32). The proportion and rank of liver cancer deaths were in third place in the three national reviews on cause of death and rose up to second place in 2020; its CMR and ASMRW did however show an increasing trend from the first time period to the second time period and a decreasing trend thereafter, in 2020. The ASMRW was 15.51/10^5^, lower than that of the national ASMRW (17.20/10^5^), which is consistent with the results of many studies (33, 34). Since the first time period, the standardized mortality rate for esophageal cancer in China has decreased (35). In our study, we found that from the first to the second time period, the ASMRW of esophageal cancer was at a high level (19.10/10^5^ and 19.59/10^5^, respectively) and remained essentially unchanged, experiencing gradual fluctuations in the two subsequent time periods. In the first and second time periods, China’s medical level was limited, so it was not possible to detect esophageal cancer through screening at an early stage; therefore, once detected, it was already at an advanced stage, which led to a higher mortality rate. With the rapid economic growth and the improvement of people’s living standard, people’s awareness of cancer prevention gradually increased. From the third time period, the public are starting to pay more attention to their lifestyles, focusing heavily on maintaining a healthy weight, not smoking, and avoiding alcohol consumption. Thus, the indicator gradually decreased.

The two cancers that had a greater impact on women were breast cancer and cervical cancer. Among them, cervical cancer ranked first in deaths during the first time period in this study. The mortality of cervical cancer and death declined during three national reviews on cause of death and slightly rebounded in 2020. Most countries showed the greatest decreasing changes in breast mortality, and some countries experienced a stable trend (36, 37). It may be possible to avoid cervical cancer by identifying and treating cervical precancerous lesions. Screening and identifying cancerous lesions early may also reduce mortality. The age-standardized mortality rates of breast cancer significantly increased in China during the third time period and 2015, but decreased in the USA, Australia, and the United Kingdom (38). This study showed that the mortality rate of breast cancer decreased from the third time period to 2020, revealing an ASMRW of 6.65/10^5^ and 4.15/10^5^, respectively. During the four periods, the lowest ASMRW of the four periods was in the second time period (2.46/10^5^). The decreasing trend in the proportion of deaths from breast cancer is consistent with the findings of some other research (39, 40). Risk factors for breast cancer are linked to China’s rapid economic development, such as the “Westernized” diet (high-fat and energy density), which increased obesity rates, physical inactivity, shortened breastfeeding durations, and later ages for first pregnancies, also significantly led to the rising mortality rate of breast cancer (41). But early screening could reduce the mortality of breast cancer, especially in women aged 50 and older (42).

Mortality reflects the frequency of death in a population pertaining to a disease, and PYLL reveals the effect of age at the time of death on life expectancy. Both can organically combine the frequency of death and life expectancy loss to reflect the burden of disease death on the population and society from different aspects (43). The burden of early death caused by cancer in the region was serious (31, 44–46). In our study, the four time periods of PYLL‰ were 9.05‰, 10.79‰, 12.1‰, and 10.38‰ from 1973 to 2020. The overall crude mortality rate of cancer in Inner Mongolia during the four periods showed an increasing trend (PPC = 1.77%, P<0.05), including an increasing trend in the crude mortality rate in males (PPC = 1.86%, P<0.05) and an increasing trend in the crude mortality rate in females (PPC = 1.68%, P<0.05).

The trend of cancer mortality among Inner Mongolia residents during the four time periods was generally on the rise with age, which is consistent with the national situation (20, 47). The mortality rates of cancer increased with age in the first time period and in 2020 for ages 10 to 84. The same trend was found for ages 0 to 74 in the second and third time periods. After peaking at age 75, it began to decline. This is slightly different from the national trend but consistent with the Jiangsu study (25, 48). The mortality rates for ages 0 to 64 in 2020 are lower than the previous three reported findings. This may be related to the continuous progress of medical care and the continuous improvement of medical examinations and screening systems nowadays. Furthermore, the increase in age-specific mortality rates afterwards compared to the previous three times may be related to the cumulative delay of 10–20 years in the accumulation of carcinogenic factors such as air pollution and smoking (49).

In summary, the three cause-of-death analyses show that the PYLL‰ of cancer is gradually increasing and that the most common type of cancer that results in death is lung cancer, but there are also other common types of cancer that occur, such as those affecting the liver, stomach, esophagus, and colon. The mortality rate of cancer in Inner Mongolia is higher in males than females, and the mortality rate increases with age. To better prevent the occurrence of cancer, we should actively encourage smoking cessation, increase the screening of cervical cancer and breast cancer for women, and actively monitor the health of older people, while also focusing on the screening of adolescents and middle-aged people. To reduce the incidence and mortality of cancer, we should strengthen research on risk factors for cancer and carry out active prevention and control in a targeted manner. This will raise the quality of life for Chinese citizens.

### 4.1 Strengths and limitations

By reviewing the relevant literature, we learned that estimating cancer mortality, its burden, and its trend over a 60-year time span in Inner Mongolia is the first to be reported. We applied CMR, ASMRC, and ASMRW to describe cancer mortality; PYLL to describe disease burden; and PPC indicators to describe trends in mortality indicators over time. However, our study also has some limitations. Firstly, different types of cancer were investigated during different survey periods. For example, cancers investigated in 1973–1975 included rectal and bowel cancers. Cancers investigated in 1990–1992 grouped colorectal and anal cancers together, and in 2020 only colorectal cancers were grouped together. This resulted in the inability to compare the mortality rates of these cancers during different periods. Second, because the time spacing of the surveys was not equidistant and the largest age group was defined as 80 years or older in 1973–1975, whereas other groups were defined as 85 years or older, they were inconsistent. For these reasons, it was not possible to use the APC (age-period-cohort) model to analyze age effects, period effects, or cohort effects. Although there were some limitations, the results of this study can still systematically and comprehensively reflect cancer mortality and its trend in Inner Mongolia.

## Data availability statement

The original contributions presented in the study are included in the article. Further inquiries can be directed to the corresponding authors.

## Author contributions

RH and ZM: Study design and manuscript writing. WN and ZL: Review and editing. BN and WK: Data collection. All authors contributed to the article and approved the submitted version.
